# Preparation of an Fe_2_Ni MOF on nickel foam as an efficient and stable electrocatalyst for the oxygen evolution reaction†

**DOI:** 10.1039/c9ra07499f

**Published:** 2019-10-18

**Authors:** Xintong Ling, Feng Du, Yintong Zhang, Yan Shen, Tao Li, Ahmed Alsaedi, Tasawar Hayat, Yong Zhou, Zhigang Zou

**Affiliations:** School of Physics, National Laboratory of Solid State Microstructures, Nanjing University Nanjing Jiangsu 210093 P. R. China zhouyong1999@nju.edu.cn; Engineering Technology Research Center of Henan Province for Solar Catalysis, School of Chemistry and Pharmaceutical Engineering, Nanyang Normal University Nanyang Henan 473061 P. R. China ltao84@163.com; Jiangsu Key Laboratory for Nano Technology, Nanjing University Nanjing Jiangsu 210093 P. R. China; Kunshan Innovation Institute of Nanjing University Kunshan Jiangsu 215347 P. R. China; College of Engineering and Applied Sciences, Nanjing University Nanjing 210093 P. R. China; Faculty of Science, King Abdulaziz University Jeddah 21589 Saudi Aribia; Department of Mathematics, Quaid-I-Azam University Islamabad 45320 Pakistan

## Abstract

Metal–organic frameworks (MOFs) as versatile templates for preparing transition metal compounds has received wide attention. Benefiting from their diversified spatial structure and controllable chemical constituents, they have become a research hotspot in the field of electrocatalytic water splitting. Herein, Fe_2_Ni-MIL-88B MOF on nickel foam (Fe_2_Ni MOF/NF) has been prepared through a one-pot method growth process. Compared with Fe MOF/NF and Ni MOF/NF, the interaction between Fe^3+^ and Ni^2+^ in Fe_2_Ni MOF/NF accelerates the electron transfer through the oxygen of the ligand, leading to increased 3d orbital electron density of Ni, which enhances the activity of the oxygen evolution reaction (OER) in alkaline solution. Fe_2_Ni MOF/NF provides a current density of 10 mA cm^−2^ at a low overpotential of 222 mV, and its Tafel slope is also very small, reaching 42.39 mV dec^−1^. The success of the present Fe_2_Ni MOF/NF catalyst is attributed to the abundant active centers, the bimetallic clusters Fe_2_Ni-MIL-88B, the positive coupling effect between Ni and Fe metal ions in the MOF, and synergistic effect between the MOF and NF. Besides, Fe_2_Ni MOF/NF possesses excellent stability over 50 h of continuous operation, providing feasibility for commercial use.

## Abstract 2

Fe_2_Ni-MIL-88B MOF on nickel foam (Fe_2_Ni MOF/NF) has excellent OER performance through a one-pot method.

## Graphical abstract

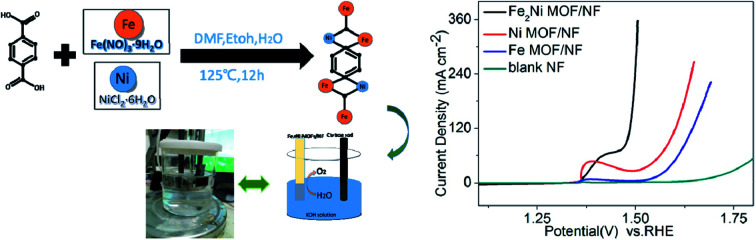

## 1. Introduction

In the last few years, with the rapid development of industrial production and the increasing population, the demand for fossil energy has increased.^1^ However, environmental pollution and global warming have driven the necessity of searching for clean energy. It is imperative to exploit and utilize renewable energy.^2–4^ Electrocatalytic water splitting is a research director with great potential at present, which includes two half-reactions: the oxygen evolution reaction (OER) and the hydrogen evolution reaction (HER). Due to involving multiple reaction steps from water to O_2_, the OER process has been considered as the bottleneck.^5^ Some noble-metal-based materials, such as RuO_2_ and IrO_2_, have relatively high electrocatalytic activity for the OER. However, their scarcity limits their large-scale application.^6,7^ The search for OER electrocatalysts with high efficiency and low cost of nonprecious metal materials has become an important task.

Transition metal compounds have been widely reported as effective OER electrocatalytic materials, including oxides, hydroxides, sulfides, nitrides and phosphides.^8–14^ Meanwhile, it is necessary to design rational engineering, in which the active sites should be fully exposed. Here, as a new nano-porous material with special structure, metal–organic framework (MOF) has some unique properties, including tunable pore sizes, large specific surface area, and perfect nanostructures. And it has obtained exciting application value in energy storage, catalysis, gas storage, *etc.*^15–21^ Generally, due to the low conductivity and stability of single metal MOF, it is rarely used as a direct electrocatalyst.^22^ The mixed metal MOF material was relatively stable and efficient. Besides, the bimetallic nickel-iron composites were identified as one of the most promising electrocatalysts for the OER, in which the interaction between metals could promote the process of electrocatalytic reaction. Therefore, nickel-iron MOF material would show great potential for development in water splitting.^23,24^

In our work, Fe and Ni-based MOF on nickel foam (NF) had been prepared by a one-pot method, which was known as Fe_2_Ni-MIL-88B MOF (Fe_2_Ni MOF). This MOF exhibited a loose nanosheet structure. And the bimetallic system accelerated the electron transfer between Fe^3+^ and Ni^2+^ through the oxygen of the ligand to increase 3d orbital electron density of Ni.^25,26^ Fe_2_Ni MOF is different from other NiFe MOF. Firstly, this MOF was a composite of Fe and Ni, not a mixture of Fe MOF and Ni MOF, so that electron transfer can be carried out between the Fe and Ni elements to improve OER activity. Secondly, Fe_2_Ni MOF's ligand was different from other Ni Fe MOF, so it has a special morphology that could increase the active site. Finally, the success of the present Fe_2_Ni MOF/NF catalyst is attributed to the synergistic effect between the MOF and NF. Specifically, compared with the monometallic MOF materials (Fe MOF/NF and Ni MOF/NF), Fe_2_Ni MOF/NF had better performance in OER. It exhibited a low overpotential of 222 mV at a current density of 10 mA cm^−2^, and a small Tafel slope of 42.39 mV dec^−1^. More importantly, Fe_2_Ni MOF/NF possessed excellent electrochemical durability of 50 hours for potential applications in the future.

## 2. Experimental

### 2.1. Preparation of Fe_2_Ni MOF/NF, Fe MOF/NF, and Ni MOF/NF

Generally, a small piece of nickel foam (NF) (1 × 6 cm) ultrasonically was washed in acetone, water, hydrochloric acid and ethanol to remove organic residues on the surface before use. The Fe_2_Ni MOF/NF was prepared by a one-pot method. First, 0.7 mmol Fe(NO)_3_·9H_2_O (0.2828 g) and 0.3 mmol NiCl_2_·6H_2_O (0.0713 g) were dissolved in *N*,*N*-dimethyl formamide (DMF). Then 1 mmol terephthalic acid (TPA), 2.5 mL deionized water and 2.5 mL ethanol was slowly added to the above solution with continuous stirring. After 30 minutes, the mixture was transferred to a 50 mL Teflon-lined stainless-steel autoclave. Then the processed NF was immersed in the mixture. The autoclave was then placed in an oven at 125 °C for 12 hours. After natural cooling, Fe_2_Ni MOF/NF was taken out and washed several times with deionized water and ethanol, and dried at 60 °C for 2 hours. Fe MOF/NF and Ni MOF/NF could be synthesized in the same way without adding NiCl_2_·6H_2_O or Fe(NO)_3_·9H_2_O, respectively.

### 2.2. Material characterization

The crystalline phase of the sample was obtained on a Rigaku Ultima III diffractometer using X-ray diffraction (XRD) with CuKα radiation (*λ* = 0.154178 nm). The scan range was 5–40° and the scan speed was 2.4 degrees per minute. The morphology and energy-dispersion X-ray spectroscopy (EDX) of the various samples was observed with scanning electron microscopy (SEM) and performed with an S-3400 N II instrument. X-ray photoelectron spectroscopy (XPS, PHI 5000 Versa Probe UlVAC-PHI, Japan) was used to obtain the XPS data of the materials, with reference to C 1s (binding energy of 284.8 eV).

### 2.3. Electrochemical measurements

The electrochemical activity of all samples was tested using a 1.0 M KOH solution in a typical three-electrode configuration using a CHI660E electrochemical workstation analyzer. The standard Ag/AgCl electrode was used as the reference electrode while the carbon rod and the synthetic sample was used as the counter electrode and the working electrode, respectively. The linear sweep voltammograms (LSV) for OER was carried out in 1.0 M KOH at a scan rate of 2 mV s^−1^. In order to eliminate the interference of the solution resistance, all measured LSV were iR-corrected. A constant current of 10 mA cm^−2^ was used for measuring long-term electrochemical stability. The recording frequency range for electrochemical impedance spectroscopy (EIS) measurement was 100 kHz to 0.01 Hz. All of the potentials herein were converted to the corresponding potentials relative to RHE using the following equation: *E*_RHE_ = *E*_Ag/AgCl_ + 0.197 + 0.0591 × pH, where *E*_RHE_ was the potential measured relative to the potential of RHE and the *E*_Ag/AgCl_ was the potential measured by the Ag/AgCl (3 M KCl) reference electrode, respectively.

## 3. Results and discussion

**Scheme 1:**
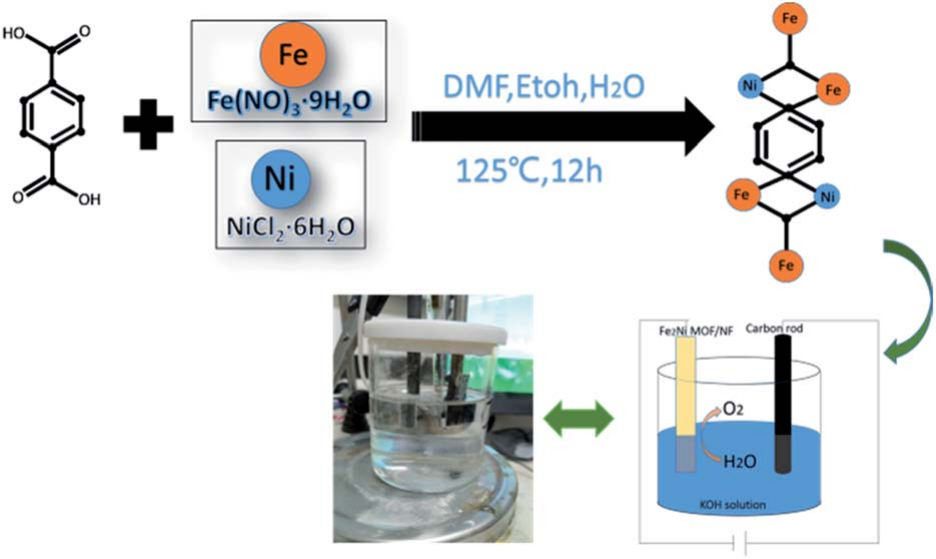
Fe_2_Ni-MIL-88B MOF on nickel foam (Fe_2_Ni MOF/NF) has been prepared through a one-pot method growth process.

**Fig. 1:**
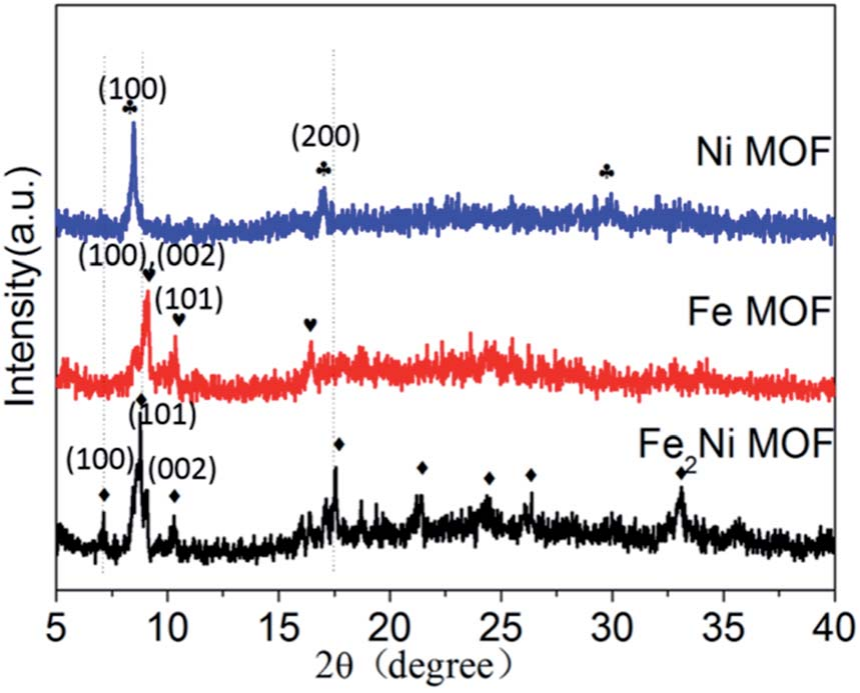
XRD patterns of Fe_2_Ni MOF/NF, Fe MOF/NF, and Ni MOF/NF.

In this report, the method for preparing Fe_2_Ni MOF grown on NF was illustrated in Scheme 1. During the solvothermal reaction, Fe_2_Ni MOF were formed in an orderly way on the surface of the NF (Fig. S1†). The composition of the three products was studied by X-ray diffraction (XRD) pattern by using the powder scraped from the corresponding substrates as shown in Fig. 1. The XRD pattern of Ni MOF has two sharp peaks at 9.3° and 18.5°, which match well with the (100) and (200) planes of [Ni_3_(OH)_2_ (C_8_H_4_O_2_)_2_(H_2_O)_4_] (CCDC no. 638866†).^27^ Fe MOF was confirmed with the diffraction pattern of Fe_3_-MIL-88B.^28^ In patterns of Fe_2_Ni MOF samples, the main diffraction peaks that appeared around 2*θ* of 7.3°, 8.9°, 9.3°, 9.9°, 16.8°, 17.7°, and 21.9° are similar to those previously reported for MIL-88B.^28^ The MIL-88B structure was established as the prominent and characteristic planes (100), (101) and (002) appeared.^29^ Additionally, we can see that the Bragg peaks of the XRD patterns show displacements. Férey *et al.* have reported that the swelling up of the MIL-88B structure causes the splitting and shifting to a low 2*θ* of the planes (100) and (101).^30^ With the presence of Ni^2+^ in the reaction solution, MIL-88B crystals were made up and the crystallinity of the material increased.

**Fig. 2:**
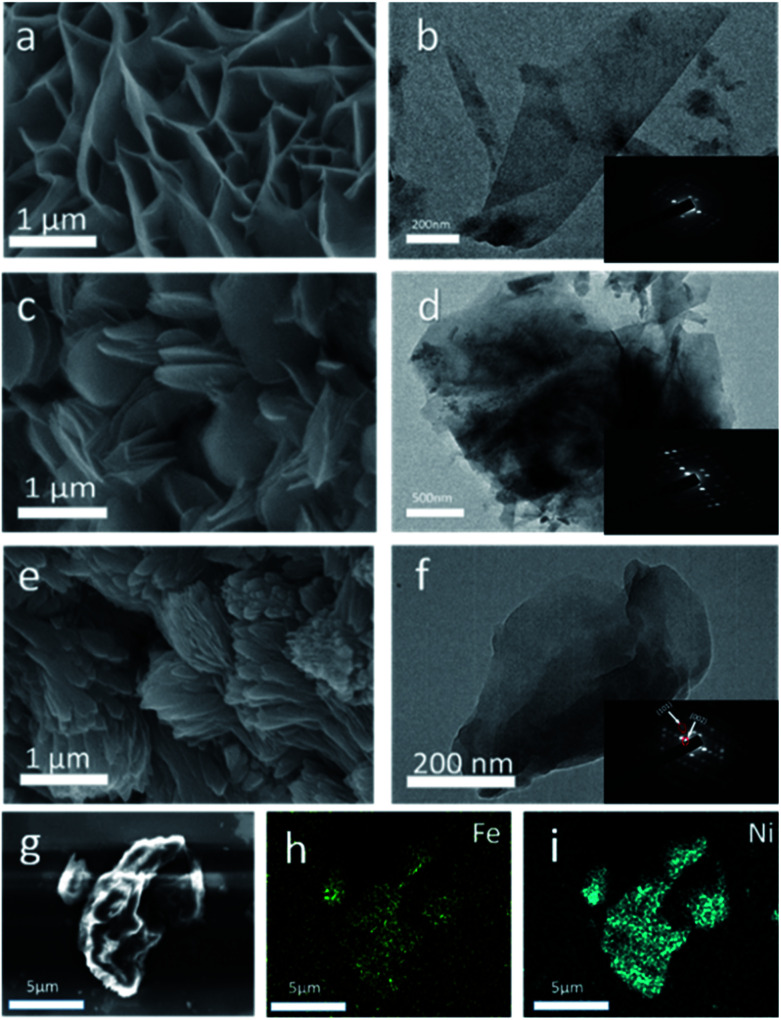
SEM images of (a) Ni MOF/NF, (c) Fe MOF/NF and (e) Fe_2_Ni MOF/NF, (b, d, and f) TEM images of Ni MOF/NF, Fe MOF/NF, and Fe_2_Ni MOF/NF. (Inset of b, d, and f): SAED pattern of Ni MOF/NF, Fe MOF/NF, and Fe_2_Ni MOF/NF. (g) SEM image of the Fe_2_Ni MOF/NF. EDS mapping images of (h) Fe and (i) Ni for the Fe_2_Ni MOF.

SEM images showed the morphology of Ni MOF/NF, Fe MOF/NF and Fe_2_Ni MOF/NF (Fig. 2a, c, and e), respectively. As shown in Fig. 2a, Ni MOF showed the structure of nanosheet arrays and the uniform coverage over the backbone surface of the NF (Fig. S2a and b†).^31^ While Fe MOF/NF had the typical shell-like morphology. Fe_2_Ni MOF/NF exhibited a spindle shape, which different from Ni MOF and Fe MOF. So they have different compositions, crystal structure, and the pore structure, their morphology is completely different. Fig. S3† reveals that the Fe_2_Ni MOF/NF still maintains its spindle shape after the stability test. And the samples with different ratios of Ni to Fe (Fig. S3a and b†), from SEM images of Fe : Ni = 3 : 7 and Fe : Ni = 5 : 5, we can find that they have sheet structure similar to Ni MOF, which imply they have a mixture of Ni MOF and Fe_2_Ni MOF. SEM image of Fe_2_Ni MOF (Fig. 2g) and high-quality EDS images into Fig. 2h and i clearly shows the presence of Fe and Ni elements in the Fe_2_Ni MOF. The TEM images of Fe_2_Ni MOF/NF, Ni MOF, and Fe MOF confirmed the respective morphological characteristics. And the selected area electron diffraction (SAED) of the three insets (Fig. 2b, d, and f) indicated the single-crystal of the samples. Additionally, from SAED of Fe_2_Ni MOF, the interplanar distances of (101) and (002) are 0.35 nm and 0.96 nm,^32,33^ which matched with the XRD results.

**Fig. 3:**
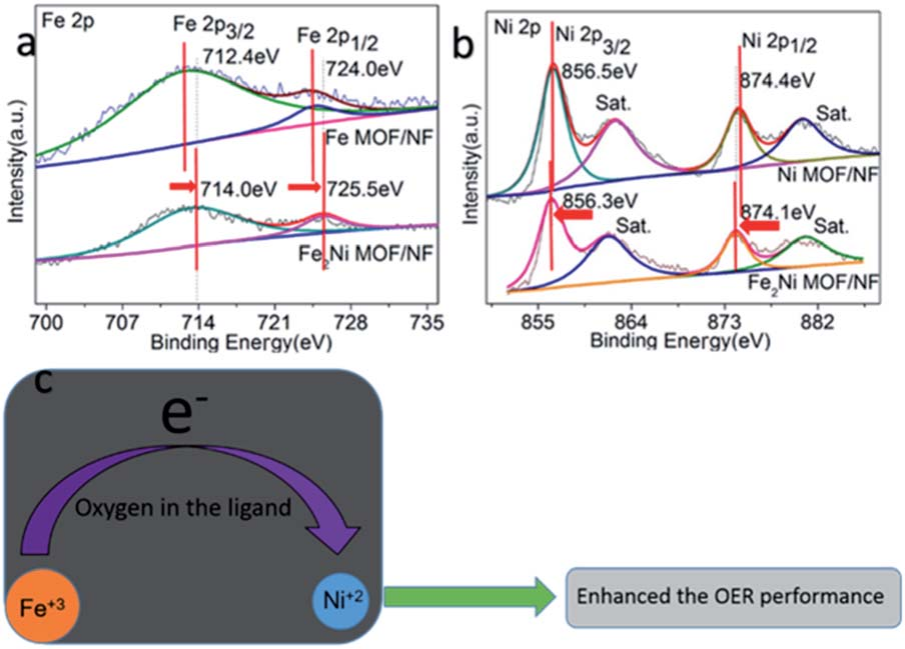
High-resolution XPS spectra of (a) Fe 2p and (b) Ni 2p of the as-prepared samples. (c) The interaction between Ni and Fe during the OER processes.

The XPS survey spectra for Fe_2_Ni MOF/NF revealed the existence of the four main constituent elements of the samples, including Fe, Ni, C, O (Fig. S4a†). The peak positions of Fe 2p and Ni 2p elements were corrected by C 1s spectra (Fig. S4b†). In the high-resolution Fe 2p spectra, the two main peaks of Fe_2_Ni MOF/NF related to the Fe 2p_3/2_ and Fe 2p_1/2_ electron configurations were located at 714 and 725.5 eV (Fig. 3a), suggesting the +3 oxidation state of Fe.^34^ The spectra of Ni 2p could be deconvoluted to exhibit binding energy peaks at around 856.3 eV and 874.1 eV for Ni 2p_3/2_ and Ni 2p_1/2_, respectively (Fig. 3b), confirming the +2 oxidation state of Ni in Fe_2_Ni MOF/NF.^35^ In comparison with Fe MOF/NF and Ni MOF/NF, the Fe 2p spectrum shifted to higher binding energy and Ni 2p spectrum shifted to lower binding energy. It implied the partial electron transfer from Fe^3+^ to Ni^2+^ through the oxygen of the ligand.^36,37^ And the Ni 2p_3/2_ and Ni 2p_1/2_ peaks in Fe_2_Ni MOF/NF were located at lower binding energy, indicating an increase of 3d orbital electron density of Ni.^38,39^ It was reported that the Ni site with electron-rich structure will increase OER activity because the OER activity of the transition metal-based material was determined by the interaction of the adsorbed OOH species with the 3d orbital of the transition metal.^40,41^ From the deconvoluted XPS of C 1s in Fig. S4b,† three peaks located at 283.1, 283.6 and 283.4 eV are assigned to adventitious hydrocarbon, C–O, and carbonate species, respectively. The O 1s XPS of the prepared samples were fitted into three peaks at 531.54, 531.99, and 531.82 eV (Fig. S4c†). Meanwhile, all the prepared samples have almost the same C 1s and O 1s binding energies, indicating that they are in the same chemical states.^42^

**Fig. 4:**
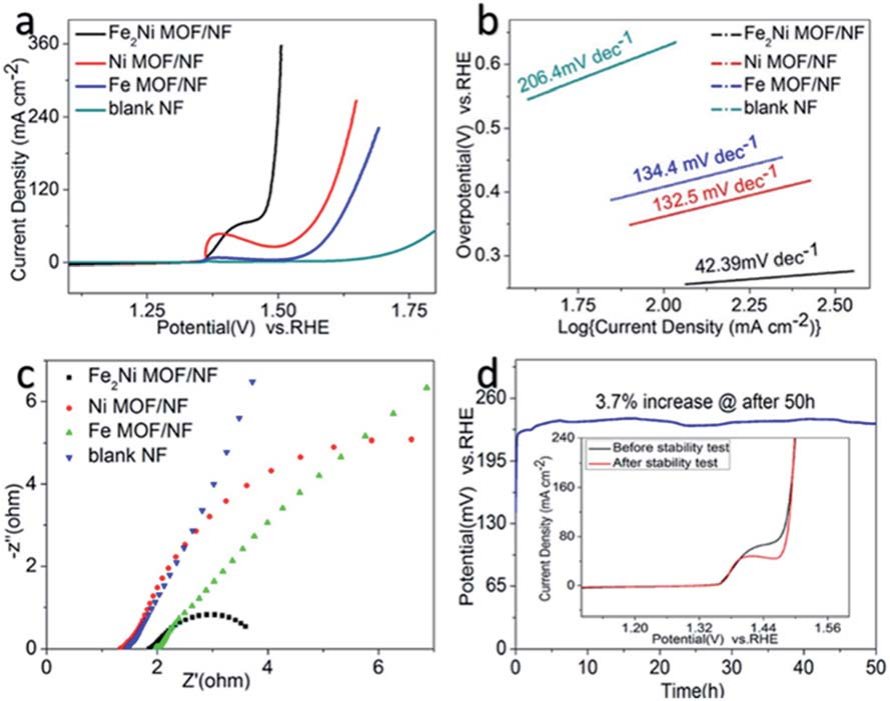
(a) CV curves, (b) Tafel plots and (c) Nyquist plots of Fe_2_Ni MOF/NF, Ni MOF/NF, Fe MOF/NF and blank NF. (d) Chronopotentiometric durability of Fe_2_Ni MOF/NF at 10 mA cm^−2^ for 50 h. (Insets of d) LSV curves of Fe_2_Ni MOF/NF before and after the long-term test.

**Fig. 5:**
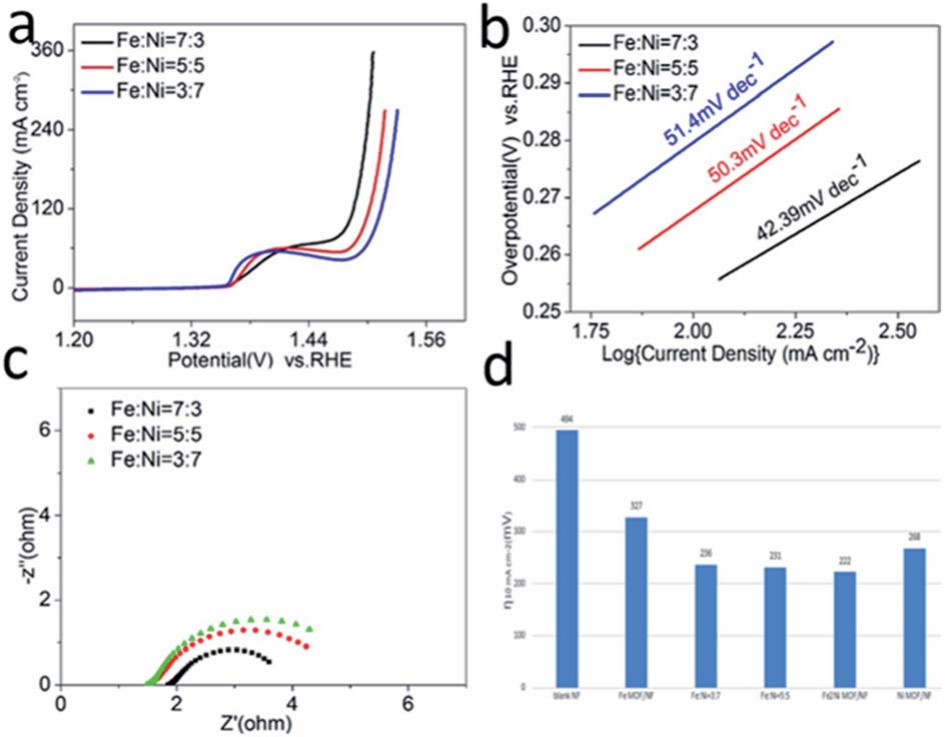
(a) LSV curves, (b) Tafel plots, and (c) Nyquist plots of Fe : Ni = 7 : 3, Fe : Ni = 5 : 5 and Fe : Ni = 3 : 7. (d) Overpotentials of Fe MOF, Ni MOF and FeNi MOF at 10 mA cm^−2^*vs.* RHE.

The OER activities of the as-synthesized samples were evaluated in 1.0 M KOH (pH = 14). Fig. 4a showed the LSV curves of Fe_2_Ni MOF/NF, Ni MOF/NF, Fe MOF/NF, and blank NF. Due to the transfer of electrons between Fe and Ni. The OER activity of the transition metal-based material was determined by the interaction of the adsorbed OOH species with the 3d orbital of the transition metal. Fe_2_Ni MOF/NF exhibited the highest OER activity. There was an oxidation peak in the process of the rising LSV curve.^43^ So we select the data of the inverse curve of CV. It only required an overpotential of 222 mV at 10 mA cm^−2^ (Fig. S5a†). The Tafel slope of the corresponding electrode could be seen from Fig. 4b, where Fe_2_Ni MOF/NF showed the smallest Tafel slope (42.39 mV dec^−1^) since the electrons in Fe are transferred to Ni. The EIS was performed at 1.63 V (*vs.* RHE) to further study the OER kinetic characteristics of the electrodes. From the Nyquist plots in Fig. 4c, benefiting from the interaction between Fe^3+^ and Ni^2+^, the charge-transfer resistance (*R*_ct_) of Fe_2_Ni MOF/NF was much less than that of Ni MOF/NF and Fe MOF/NF. Fig. 5a–d expressed the MOF of Fe : Ni = 7 : 3 performed as the best OER activity compared to Fe : Ni = 3 : 7 and Fe : Ni = 5 : 5. Because the electrons in Fe are fully transferred to Ni when the Ratio was 7 : 3. For commercial water splitting, the electrode material should have excellent long-term stability. Here, the Fe_2_Ni MOF/NF electrode was tested with chronopotentiometry (*V*–*t*) measurements, the achieved current densities increased by only 3.7% compared with the initial after a 50 h continuous operation (Fig. 4d). In addition, there was no obvious attenuation between the LSV curves obtained before and after the stability test, indicating that the Fe_2_Ni MOF/NF not only had good OER activity but also provided excellent electrochemical stability (insets of Fig. 4d). The activity comparison with recently reported MOF catalysts was conducted in Table S3.†

## 4. Conclusions

In conclusion, an efficient and durable Fe_2_Ni MOF/NF was prepared by a one-pot method. Compared to the Fe MOF/NF and Ni MOF/NF, the Fe_2_Ni MOF/NF exhibited more effective OER activity owing to the interaction between Fe^3+^ and Ni^2+^. The Ni site with an electron-rich structure will increase OER activity because the OER activity of the transition metal-based material was determined by the interaction of the adsorbed OOH species with the 3d orbital of the transition metal. As a result, Fe_2_Ni MOF/NF had a low OER overpotential of 222 mV at 10 mA cm^−2^ and a small Tafel slope of 42.39 mV dec^−1^. In addition, its long-term stability provided the possibility for commercial water splitting applications.

## Conflicts of interest

There are no conflicts to declare.

## Supplementary Material

RA-009-C9RA07499F-s001
