# Supplementary material for: Preparation of an Fe2Ni MOF on nickel foam as an efficient and stable electrocatalyst for the oxygen evolution reaction
Source: RSC Adv. 2019 Oct 18;9(57):33558–62. doi: 10.1039/c9ra07499f (PMC9073369; doi:10.1039/c9ra07499f)
Supplement: RA-009-C9RA07499F-s001 [file RA-009-C9RA07499F-s001.pdf]

Electronic Supplementary Material (ESI) for RSC Advances.
This journal is © The Royal Society of Chemistry 2019

Supporting Information

Preparation of Fe_2Ni MOF on nickel foam as an efficient and stable electrocatalyst for the oxygen evolution reaction

Xintong Ling,[†, a, c] Feng Du,[†, a, c] Yintong Zhang,[a, c] Yan Shen,[c, e] Tao Li,[*, b] Ahmed Alsaedi,[f] Tasawar Hayat,[f, g] Yong Zhou,[*, a, c, d] and Zhigang Zou[a, c, d]

[a] School of Physics, National Laboratory of Solid State Microstructures, Nanjing University, Nanjing, Jiangsu 210093, P. R. China
b] Engineering Technology Research Center of Henan Province for Solar Catalysis, School of Chemistry and Pharmaceutical Engineering, Nanyang Normal University, Nanyang, Henan 473061, P. R. China
[c] Jiangsu Key Laboratory for Nano Technology, Nanjing University, Nanjing, Jiangsu 210093, P. R. China
[d] Kunshan Innovation Institute of Nanjing University, Kunshan, Jiangsu 215347, P. R. China
[e] College of Engineering and Applied Sciences, Nanjing University, Nanjing 210093, P. R. China
[f] Faculty of Science, King Abdulaziz University, Jeddah 21589, Saudi Aribia
[g] Department of Mathematics, Quaid-I-Azam University, Islamabad 45320, Pakistan
† Ling and Du contributed equally to this work

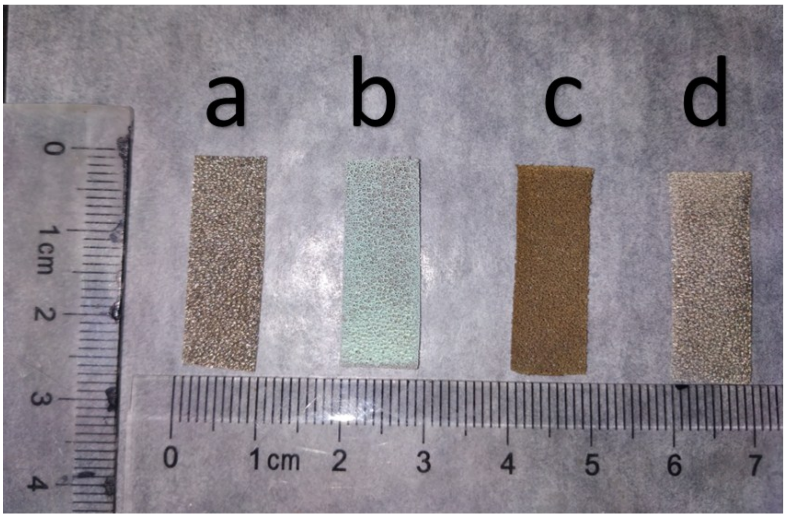

Fig. S1 Photos of nickel foams: (a) blank NF, (b) Ni MOF/NF, (c) Fe_2Ni MOF/NF and (d) Fe MOF/NF.

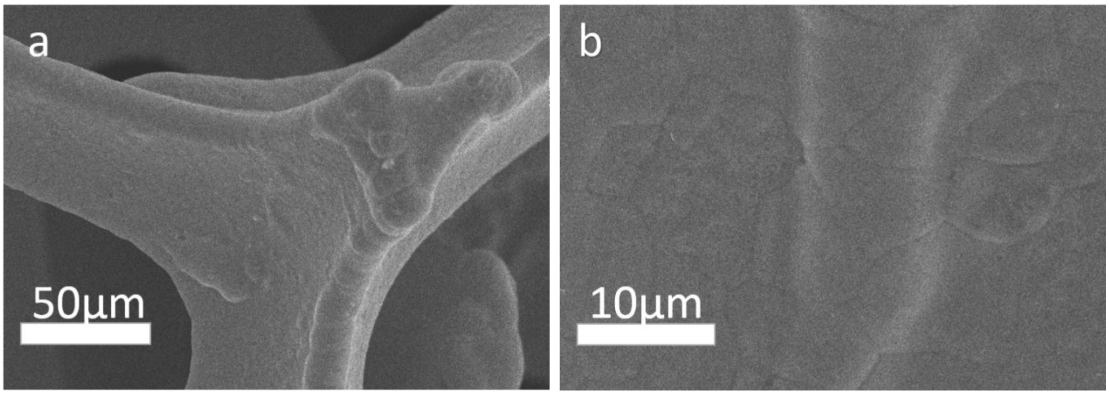

Fig. S2 SEM images of Ni foam(a,b).

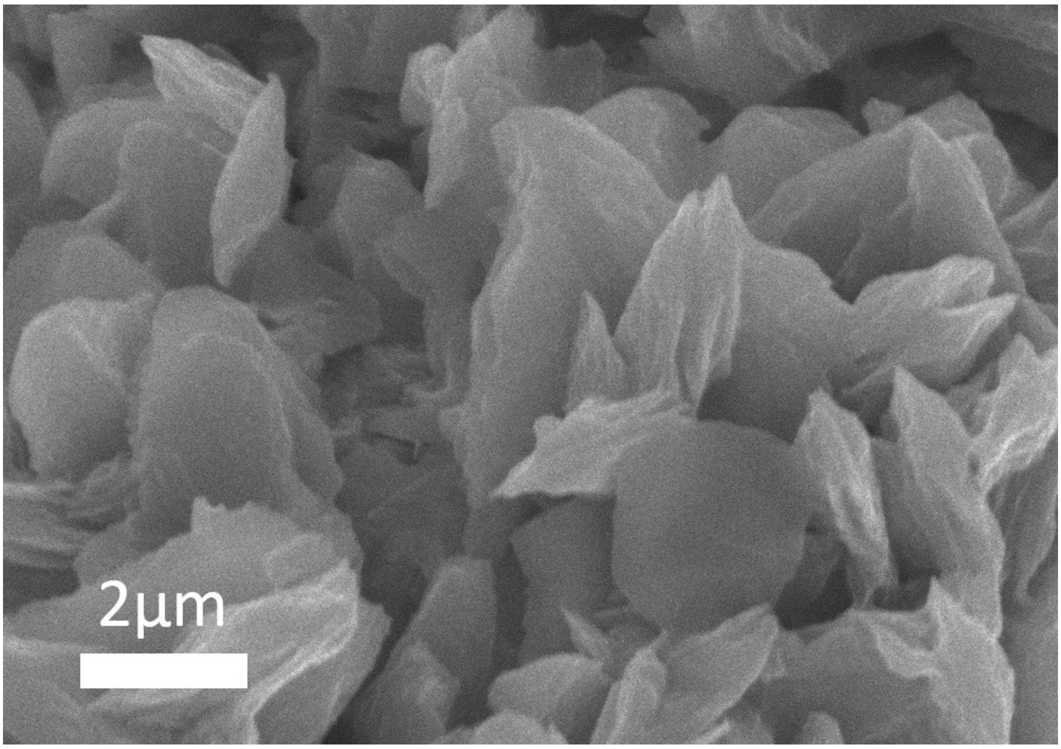

Fig. S3 SEM image of Fe_2Ni MOF/NF after stability test.

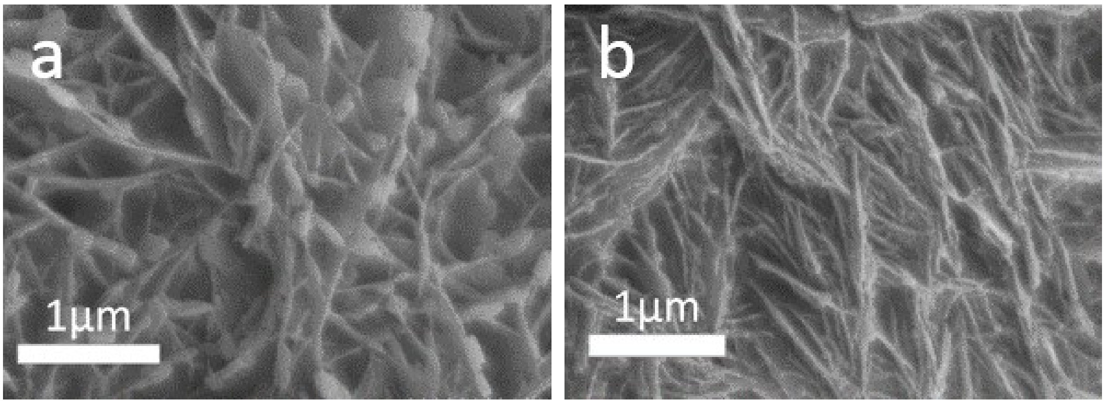

Fig. S4 (a) SEM images of Fe:Ni=3:7 and (b) Fe:Ni=5:5.

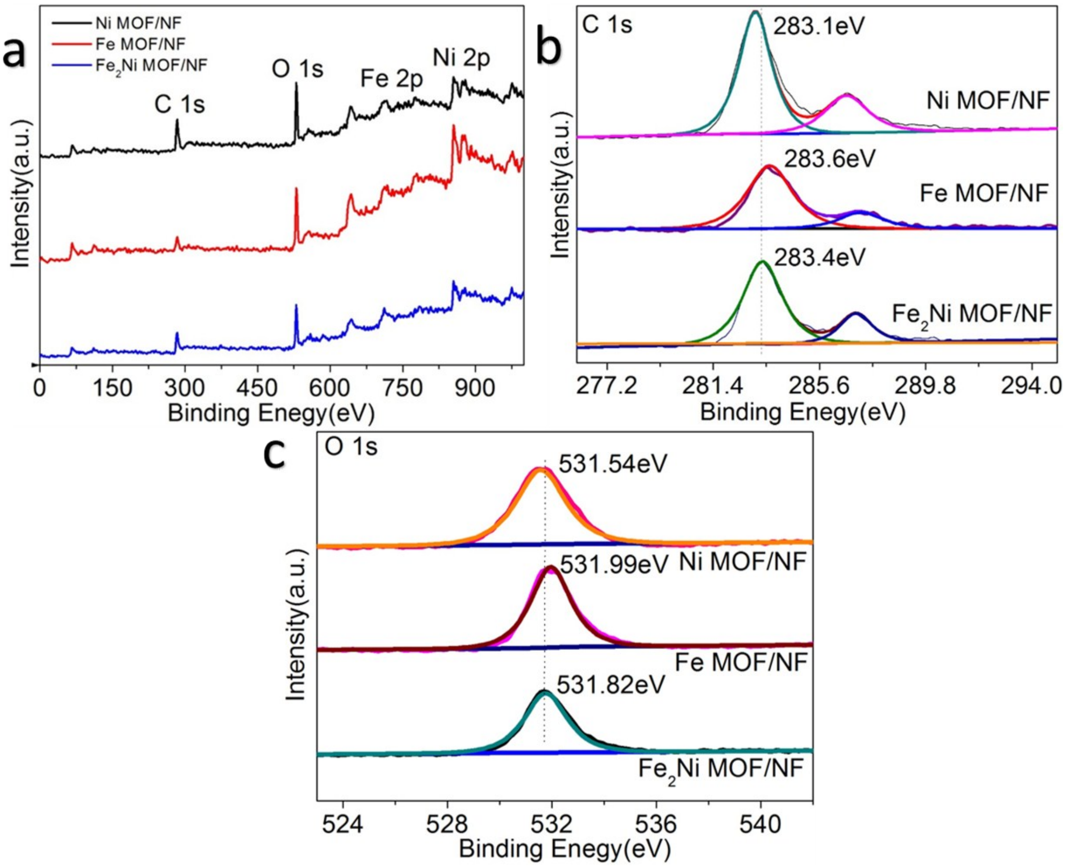

Fig. S5 (a) Fully measured XPS spectra of Fe_2Ni MOF/NF, Ni MOF/NF and Fe MOF/NF, High-resolution XPS spectra of (b) C 1s and (c) O 1s of the as-prepared samples.

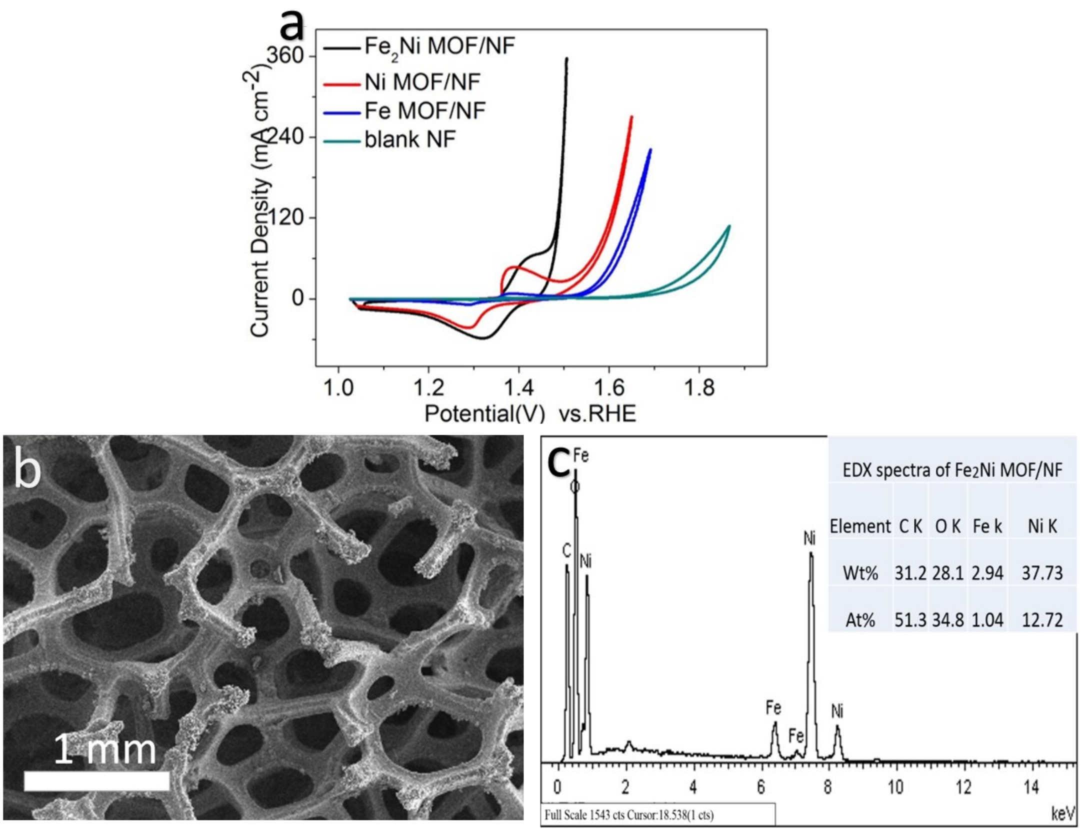

Fig. S6 (a) CV curves of Fe_2Ni MOF/NF, Ni MOF/NF, Fe MOF/NF and blank NF , (b) SEM images with corresponding (c) EDX results of Fe_2Ni MOF/NF, (Inset of c) : table of elemental composition.

Table S1 OER performances for blank NF, Fe MOF/NF, Ni MOF/NF and Fe_2Ni MOF/NF.

Catalysts	η(at 10 mA cm 2)	Tafel slope
blank NF	494 mV	206.4 mV dec^{-1}
Fe MOF/NF	327 mV	134.4 mV dec^{-1}
Ni MOF/NF	268 mV	132.5 mV dec^{-1}
Fe_2Ni MOF/NF	222 mV	42.4 mV dec^{-1}

Table S2 OER performances for Fe:Ni=3:7, Fe:Ni=5:5 and Fe:Ni=7:3.

Catalysts	η(at 10 mA cm 2)	Tafel slope
Fe:Ni=3:7	236 mV	51.4 mV dec^{-1}
Fe:Ni=5:5	231 mV	50.3 mV dec^{-1}
Fe:Ni=7:3	222 mV	42.4 mV dec^{-1}

Table S3 Comparison of OER performance in alkaline media with other reported non-precious electrocatalysts.

Catalysts	η (mV @ mA cm-2)	Tafel slope (mV dec-1)	Ref
Fe_2Ni MOF/NF	222@10	42.4	This work
MNF-MOFs/NF	235@50	55.4	Nano Energy. 2019, 57 1–13.
$Fe_{0.1}$-Ni-MOF/NF	243@50	69.8	J. Mater. Chem. A. 2019, 7, 8771.
NFN-MOF/NF	240@10	58.8	Adv. Energy Mater. 2018, 8, 1801065.
Fe-MOF/NF	240@50	72	Inorg. Chem. Front. 2018,51, 405.
Ni-MOF/NF	320@100	123	Inorg. Chem. Front. 2018, 5, 1570.
FeNi-DOBDC	270@50	34	ACS Sustainable Chem. Eng. 2019, 7, 9743−9749
NiFe-MOF-74	223@10	71.6	Chem. Commun., 2018, 54, 7046—7049
NiFe-NFF	227@10	38.9	Adv. Funct. Mater. 2019, 29, 1807418
Co_3S_4/EC-MOF	226@10	132	Adv. Mater. 2019, 31, 1806672
Nix$Co_{3-x}O_4$/NF	287@10	88	Nanoscale, 2019,11, 11765-11773
FeNi-MOF	270@50	49	ACS Sustainable Chem. Eng. 2019, 7, 9743−9749
$(Ni2Co1)_{0.925}Fe_{0.075}$-MOF-NF	257@10	41.3	Adv. Mater. 2019, 31, 1901139
Ni–Fe–MOF NSs	221@10	56.0	Angew. Chem. Int. Ed. 2019, 58, 7051 – 7056
CoNi-MOF/rGO	318@10	48	ACS Appl. Mater. Interfaces 2019, 11, 15662−15669
EC-MOF	215@20	37	ACS Appl. Energy Mater. 2019, 2, 2138−2148
(Co,Ni)Se_2@NiFe LDH	277@10	75	ACS Appl. Mater. Interfaces 2019, 11, 8106−8114
